# Oat Beta-Glucan as a Metabolic Regulator in Early Stage of Colorectal Cancer—A Model Study on Azoxymethane-Treated Rats

**DOI:** 10.3390/ijms25094635

**Published:** 2024-04-24

**Authors:** Jacek Wilczak, Adam Prostek, Katarzyna Dziendzikowska, Małgorzata Gajewska, Łukasz Kopiasz, Joanna Harasym, Michał Oczkowski, Joanna Gromadzka-Ostrowska

**Affiliations:** 1 Department of Physiological Sciences, Institute of Veterinary Medicine, Warsaw University of Life Sciences, 02-776 Warsaw, Poland; adam_prostek@sggw.edu.pl (A.P.); malgorzata_gajewska@sggw.edu.pl (M.G.); 2 Department of Dietetics, Institute of Human Nutrition Sciences, Warsaw University of Life Sciences, 02-776 Warsaw, Poland; katarzyna_dziendzikowska@sggw.edu.pl (K.D.); lukasz_kopiasz@sggw.edu.pl (Ł.K.); michal_oczkowski@sggw.edu.pl (M.O.); joanna_gromadzka-ostrowska@sggw.edu.pl (J.G.-O.); 3 Department of Biotechnology and Food Analysis, Wroclaw University of Economics and Business, 53-345 Wroclaw, Poland; joanna.harasym@ue.wroc.pl

**Keywords:** azoxymethane, colorectal cancer, interleukins, metabolome, oat beta-glucan, redox status

## Abstract

Factors that reduce the risk of developing colorectal cancer include biologically active substances. In our previous research, we demonstrated the anti-inflammatory, immunomodulatory, and antioxidant effects of oat beta-glucans in gastrointestinal disease models. The aim of this study was to investigate the effect of an 8-week consumption of a diet supplemented with low-molar-mass oat beta-glucan in two doses on the antioxidant potential, inflammatory parameters, and colonic metabolomic profile in azoxymethane(AOM)-induced early-stage colorectal cancer in the large intestine wall of rats. The results showed a statistically significant effect of AOM leading to the development of neoplastic changes in the colon. Consumption of beta-glucans induced changes in colonic antioxidant potential parameters, including an increase in total antioxidant status, a decrease in the superoxide dismutase (SOD) activity, and a reduction in thiobarbituric acid reactive substance (TBARS) concentration. In addition, beta-glucans decreased the levels of pro-inflammatory interleukins (IL-1α, IL-1β, IL-12) and C-reactive protein (CRP) while increasing the concentration of IL-10. Metabolomic studies confirmed the efficacy of oat beta-glucans in the AOM-induced early-stage colon cancer model by increasing the levels of metabolites involved in metabolic pathways, such as amino acids, purine, biotin, and folate. In conclusion, these results suggest a wide range of mechanisms involved in altering colonic metabolism during the early stage of carcinogenesis and a strong influence of low-molar-mass oat beta-glucan, administered as dietary supplement, in modulating these mechanisms.

## 1. Introduction

Colorectal cancer (CRC) ranks as the third most diagnosed type of cancer and the second leading cause of cancer-related deaths worldwide [1]. Primary risk factors for CRC development include intestinal inflammation (typically non-specific), older age, and environmental factors, including alcohol consumption, smoking, obesity, and diet type. Although early stages of CRC often remain asymptomatic, early diagnosis is associated with a good prognosis of approximately 90% of 5-year survival rate [2]. Recent studies suggest that diet (its quality and the content of potentially carcinogenic ingredients) plays a crucial role in the initiation of inflammatory processes that lead to carcinogenesis. Meta-analysis performed by Shivappa and co-workers has shown a direct link between the consumption of certain dietary products and an increased risk of CRC development [3]. On the other hand, various factors, including regular physical activity a diet rich in fruits, vegetables, fibre, folic acid, calcium, dairy products, vitamin D, vitamin B6, magnesium, garlic, and regular fish intake, have been associated with a reduction in CRC rates [4]. Specific dietary components, such as polyphenols, prebiotic fibre fractions, polyunsaturated n-3 fatty acids, known for their antioxidative and anti-inflammatory properties, may exert an inhibitory effect on CRC development [5].

Among the dietary components that show potential direct anti-inflammatory and indirect antioxidative effects are beta-glucans. Beta-glucans are a group of compounds composed of D-glucose molecules linked by beta-glycosidic bonds, forming non-starch polysaccharides that belong to the dietary fibre fraction. They serve as a structural components in the cell walls of fungi, yeasts, and marine algae, as well as the aleurone layer and endosperm of certain cereals. The structure of beta-glucans varies depending on their source of origin, which in turn leads to their different biological functions. Cereal beta-glucans (including those isolated from oats), unlike mushroom, yeast, and seaweed beta-glucans, form long, slightly branched chains consisting of β-D-glucopyranose monomers linked mainly via β-1,4-glycosidic bonds and, to a lesser extent, β-1,3-glycosides. This unique structure renders oat beta-glucans well soluble in water, forming sticky gels. Among the numerous properties of beta-glucans that directly affect gastrointestinal tract functions, their anti-inflammatory and prebiotic properties are of most interest. Our previous research demonstrated the inhibitory effect of oat beta-glucans on LPS-induced inflammatory process in rats’ colon [5,6]. In these studies, the effect of high- and low-molar-mass oat beta-glucans was investigated, and our results showed that the low-molar-mass beta-glucan had stronger anti-inflammatory properties [5,6]. Therefore, in the current study, we focused on oat beta-glucan with a low molar mass (5.2 × 10^4^ g/mol) and purity of 99.3%.

To demonstrate the anti-carcinogenic properties of the low-molar-mass oat beta-glucan, we used an experimental model of azoxymethane (AOM)-induced early-stage colorectal cancer in rats. AOM and its final metabolites of 1,2-dimethylhydrazine (DMH) are responsible for the methylation of the DNA nitrogenous bases in various cells, including the epithelial cells of colonic crypts. This process leads to colon cell apoptosis and an increased mutagenicity of colonic epithelial cells [7,8,9,10]. In the present study, we determined the levels of selected pro- and anti-inflammatory cytokines (IL-1α, IL-1β, IL-12, IL-10, as well as C reactive protein), markers of the redox status (glutathione reductase, glutathione peroxidase, superoxide dismutase, and total antioxidant status), as well as the metabolomic profile in the large intestine tissue of rats with AOM-induced early stage of colorectal cancer. The rats were fed either a feed supplemented with the low-molar-mass oat beta-glucan (OBG) at the level of 1% or 3% or a non-supplemented feed. This comprehensive approach was adopted to explore the possible mechanisms underlying the protective effect of oat beta-glucans against carcinogenesis in the colon.

## 2. Results

### 2.1. Colorectum Histopathological Changes

**Figure 1:**
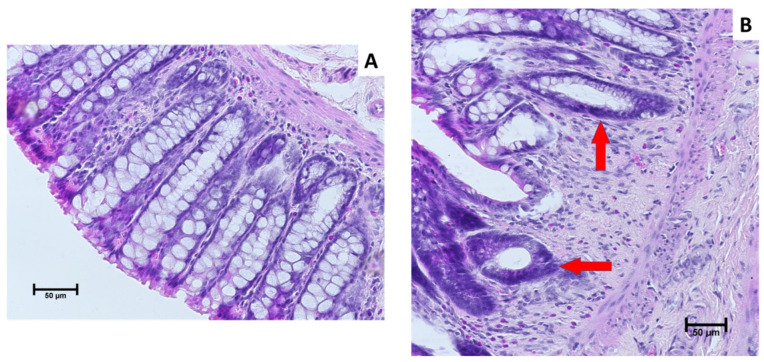
Microscopic images of colon epithelium of experimental rats. (**A**) normal colon epithelium; (**B**) colon epithelium with aberrant crypts and hyperplasia (red arrows).

Histopathological examination of colorectal specimens showed pronounced changes in the epithelium, including the presence of aberrant crypts and hyperplasia (Figure 1B), observed mainly in rats from the CRC group without dietary intervention, which confirms the effectiveness of the research model used. In control animals, regardless of the type of diet consumed, and in most of the rats from groups CRC_BG_1 and CRC_BG_3, the colorectal epithelium showed normal morphology without microscopic signs of abnormalities (Figure 1A). A detailed description of the analysis of histological changes can be found in our previous publication [11].

### 2.2. The Redox Balance of the Colorectal Tissue

**Figure 2:**
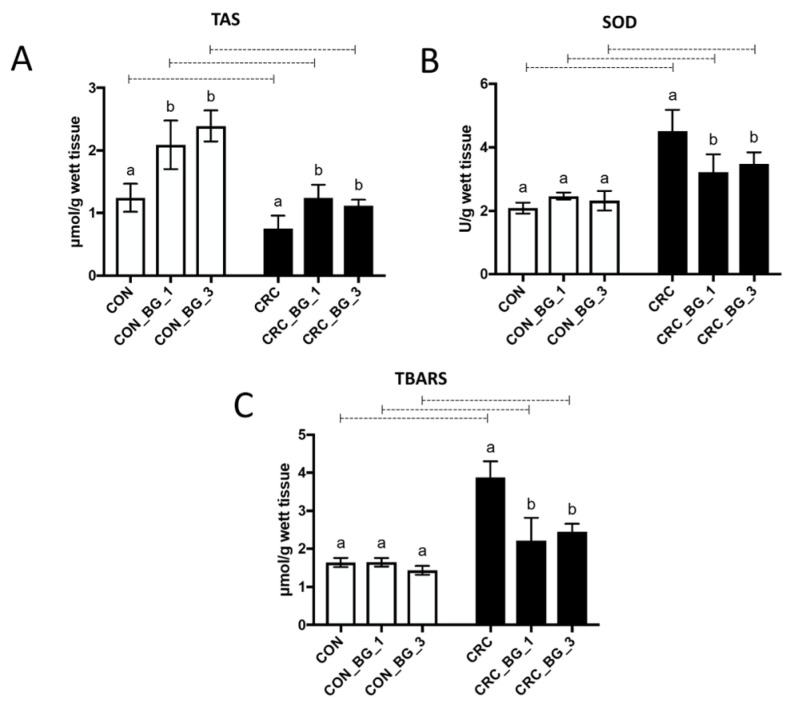
Redox status of the colorectal tissue: total antioxidant status (TAS) (**A**), superoxide dismutase activity (SOD) (**B**), and thiobarbituric acid reactive substances (TBARSs) (**C**) in colorectal tissue of rats from control (CON) and AOM-induced colorectal cancer (CRC) groups fed a diet with 1% or 3% of oat beta-glucan (BG_1 and BG_3, respectively) or control diet. Results are presented as means ± standard deviation. Statistically significant differences between groups and within groups were evaluated using two-way ANOVA with Tukey’s post hoc test. Means followed by a common letter are not significantly different at the 5% level of significance (*p* < 0.05) when the comparison is made among dietary subgroups in the control/CRC group. Connecting dotted line is placed above bars that represent statistically different means between the control and CRC groups on the same feed at *p* < 0.05.

The analyses indicated significant changes in the redox balance in the colorectum of the examined animals. AOM treatment resulted in a statistically significant decrease in TAS level along with a simultaneous increase in SOD activity and TBARS concentration in rats from the CRC group in comparison to animals from the CON group. On the other hand, in the CON_BG_1 and CON_BG_3 groups, the SOD activity and TBARS concentration were at the same levels as those in the corresponding control group, although TAS levels were increased in comparison to those in the control group (CON). In animals with CRC, the 8-week consumption of feed supplemented with 1% or 3% OBG (CRC_BG_1 and CRC_BG_3 groups) significantly altered all parameters when compared with corresponding CON groups. TAS values in CRC_BG_1 and CRC_BG_3 groups reached levels observed in animals from the CON group (Figure 2).

An analysis of all markers related to glutathione metabolism (Table 1) showed that AOM administration caused a decrease in reduced gluthatione (GSH) and an increase in the oxidized gluthatione (GSSG) concentration, which resulted in the lowest GSH/GSSG ratio in the colorectal tissue of rats from the CRC group in comparison to other tested groups. In the colorectal tissue of rats from the CON group, the value of GSH/GSSG ratio was higher (0.13 ± 0.02 vs. 0.68 ± 0.07). Dietary intervention with OBG, regardless of the dose used, increased the GSH/GSSG ratio to the value of approximately 0.31 in the case of animals from the CRC_BG_1 and CRC_BG_3 groups. The rate of conversion of GSSG to GSH is a consequence of the activity of glutathione reductase (GR). Its activity did not change in control animals fed a diet containing OBG, regardless of its amount in the diet. However, the activity of GR was decreased during AOM-induced CRC. Supplementation of diet with OBG significantly increased the GR activity in comparison to the CRC group, but this effect was observed only in the case of 1% OBG dietary dose (group CRC_BG_1). In addition, a significant increase in glutathione peroxidase (GPx) activity was observed in CRC groups. Only in rats from the CRC_BG_1 group was a significant decrease in the activity of GPx was observed; however, this value did not reach the level characteristic for control animals (CON_BG_1 and CON_BG_3 groups).

### 2.3. Immunological Parameters in Colorectal Tissue Samples

**Figure 3:**
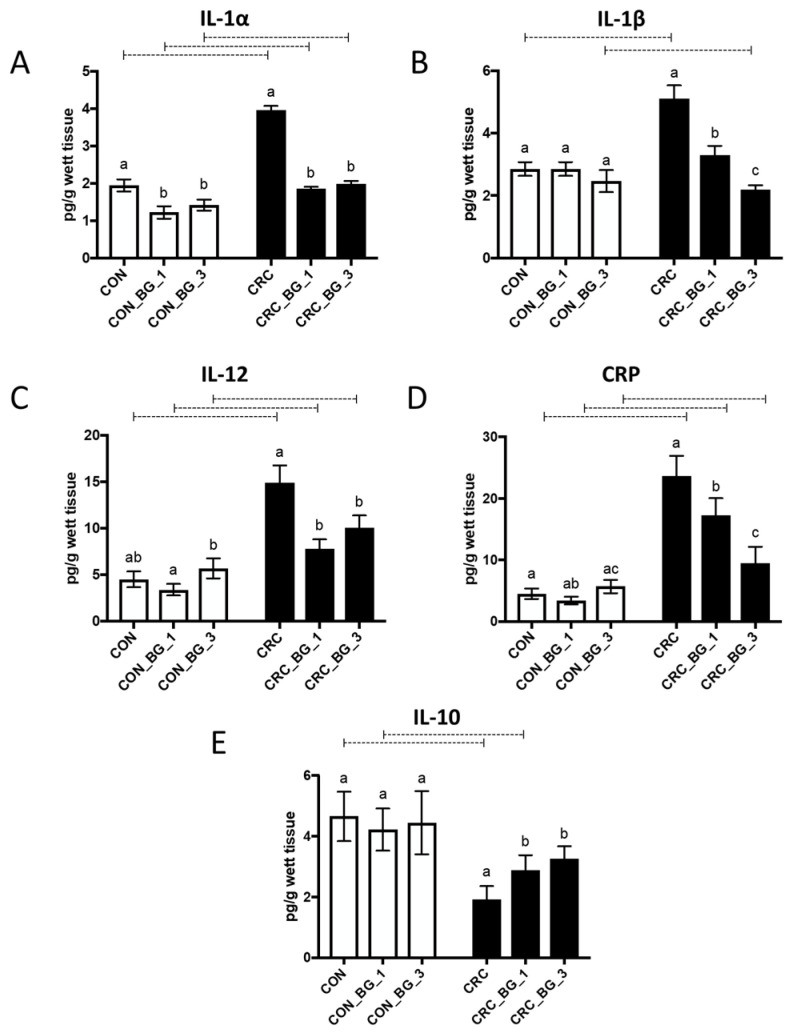
Immunological parameters in colorectal tissue: levels of interleukin 1alpha (IL-1α) (**A**), Interleukin 1beta (IL-1β) (**B**), interleukin 12 (IL-12) (**C**), C-reactive protein (CRP) (**D**), and interleukin 10 (IL-10) (**E**) in colorectal tissue of rats from control (CON) and AOM-induced colorectal cancer (CRC) groups fed a diet with 1% or 3% of oat beta-glucan (BG_1 and BG_3, respectively) or control diet. Results are presented as means ± standard deviation. Statistically significant differences between groups and within groups were evaluated using two-way ANOVA with Tukey’s post hoc test. Means followed by a common letter are not significantly different at the 5% level of significance (*p* < 0.05) when the comparison is made among dietary subgroups in the control/CRC group. Connecting dotted line is placed above bars that represent statistically different means between the control and CRC groups on the same feed at *p* < 0.05.

The induction of the early stage of CRC in rats by AOM (CRC group) caused an increase in the concentration of pro-inflammatory interleukins and CRP protein, accompanied by a statistically significant decrease in the concentration of the anti-inflammatory cytokine IL-10 (Figure 3). The intake of feed supplemented with OBG had the greatest effect on changing the inflammatory profile of the colorectal tissue, especially in animals from the CRC groups. The concentrations of both pro-inflammatory interleukins IL-1α and IL-1β, in CRC_BG_1 and CRC_BG_3 groups, reached values similar to those in the control group (CON), suggesting that the consumption of feed with OBG lowered the concentration of those interleukins to physiological values (Figure 3A,B). A similar trend was observed in the case of IL-10. The level of anti-inflammatory cytokine IL-10 was increased in the colorectal tissue of CRC rats receiving diet with OBG in comparison to corresponding CRC group (Figure 3E). Consumption of feed supplemented with 1% or 3% of OBG (CRC_BG_1 and CRC_BG_3) caused a significant reduction in the IL-12 and CRP levels; however, the concentration of these proteins did not reach the values observed in control animals (Figure 3C,D). In the case of CRP, the strongest effect was noted when rats were fed a diet supplemented with 3% OBG (Figure 3D).

### 2.4. The Metabolomic Profile of Colorectal Tissue

To determine the changes in metabolomic profiles of CRC rats under the influence of consumption of feed supplemented with two different doses of OBG, metabolomic data from the following group comparisons were analysed: CRC vs. CON, CRC vs. CRC_BG_1 and CRC vs. CRC_BG_3. This approach allowed a precise comparison of the metabolomes of the large intestine under the influence of tested factors, including AOM effects and dietary intervention. In addition, it facilitated the determination of the metabolic pathways connected with the above factors. The final effect of such comparison was the selection of metabolites that generated the most pronounced and statistically significant differences between the experimental groups.

Data were mean-centred using scaling and principal component analysis (PCA) to analyse the metabolomic data. A *p* value at ≤0.05 was considered statistically significant. Metabolites that differed among tested groups were subjected to pathway analysis by MetaboAnalyst 5.0, which combined results from powerful pathway enrichment analysis with the pathways’ topology analysis. The identified metabolites were then mapped to the KEGG pathway for biological interpretation to achieve a higher-level understanding of systemic functions.

**Figure 4:**
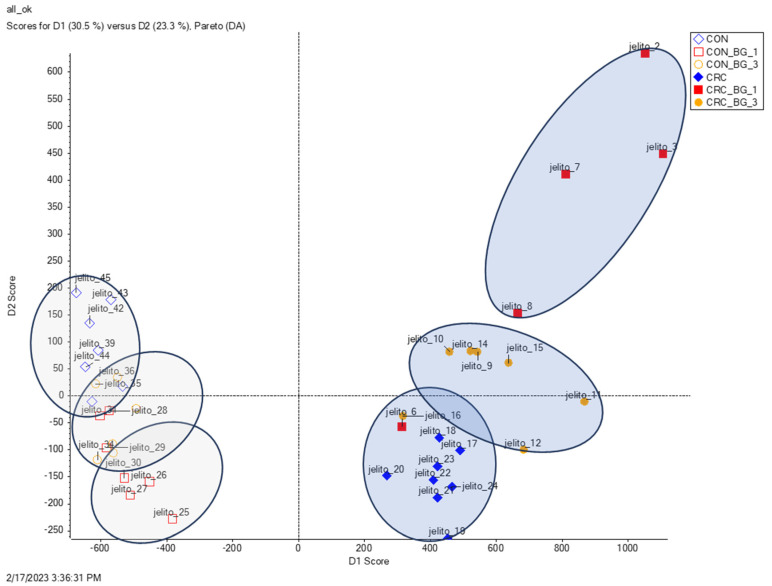
Principal components analysis (PCA) of colorectal tissue metabolome of rats from control (CON) and AOM-induced colorectal cancer (CRC) groups fed a diet with 1% or 3% of oat beta-glucan (BG_1 and BG_3, respectively) or control diet. Areas with a grey background represent metabolomes characteristic for control groups (CON), whereas areas with blue background represent metabolomes characteristic for CRC groups.

A PCA analysis (Figure 4) showed that the metabolomic profile in the colorectal tissue samples of rats was statistically significantly affected by both the AOM treatment (CON vs. CRC) and by consumption the feed supplemented with different doses of OBG. The blocked diagrams show the separation of metabolomes of groups CON and CRC (Figure 4). Homogeneity within the control groups (non-supplemented and supplemented with OBG) can be noticed. The consumption of 1% or 3% OBG with the feed did not cause pronounced differences in the metabolomes of control animals.

This difference in metabolomic profiles was also reflected in the number of differentiating metabolites detected in the investigated groups. Within all CON groups, 465 differential metabolites were identified, whereas in three CRC groups, the number of characteristic metabolites amounted to 1435. Therefore, a more detailed analysis was conducted to examine the differences in metabolomes of the following experimental groups: CRC vs. CON, CRC vs. CRC_1BG, CRC vs. CRC_3BG (Supplementary Data, Tables S1–S3). Supplementary Table S1 presents metabolic pathways in which the metabolites differed between the CRC and CON groups. The following metabolic pathways were found: metabolism of butanoate, valine, leucine and isoleucine degradation, metabolism of lysine and tryptophan, fatty acids degradation and metabolism, purine metabolism, sphingolipid metabolism, one carbon pool by folate, and others.

Metabolites that differed significantly between CRC and CRC_BG_1 groups (fed non-supplemented versus supplemented with 1% OBG diets) represented the following metabolic pathways: arachidonic acid, linoleate, vitamin A (retinol), glycerophospholipid, porphyrin metabolism, prostaglandin formation from arachidonate, leukotriene metabolism, and C21-steroid hormone biosynthesis and metabolism (Supplementary Table S2a). When the amount of OBG added to the feed was increased to 3%, the metabolites that differed significantly between CRC and CRC_BG_3 groups represented the following metabolic pathways: aspartate and asparagine metabolism, methionine and cysteine metabolism, mono- and di-unsaturated fatty acid beta-oxidation, leukotriene, linoleate metabolism, omega-3 fatty acids metabolism, prostaglandin formation from arachidonate, C21-steroid hormone biosynthesis and metabolism, androgen and oestrogen biosynthesis and metabolism, porphyrin metabolism, and others (Supplementary Table S3a). Significantly (*p* < 0.05) regulated metabolites detected in the colorectal tissues of rats from analysed experimental groups are presented in Supplementary Tables S1b, S2b, and S3b.

**Figure 5:**
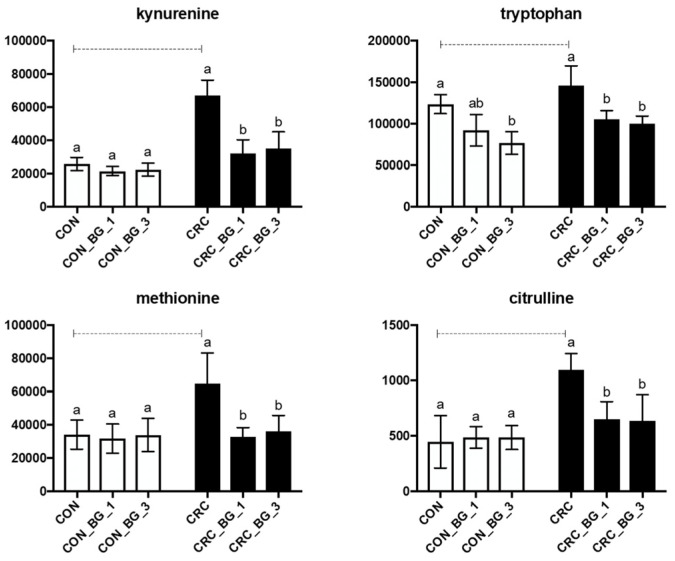
Sum of m/z metabolite counts of kynurenine, methionine, citrulline, tryptophan in colorectal tissue of rats from control (CON) and AOM-induced colorectal cancer (CRC) groups fed a diet with 1% or 3% of oat beta-glucan (BG_1 and BG_3, respectively) or control diet. Results are presented as means ± standard deviation. Statistically significant differences between groups and within groups were evaluated using two-way ANOVA with Tukey’s post hoc test. Means followed by a common letter are not significantly different at the 5% level of significance (*p* < 0.05) when the comparison is made among dietary subgroups in the control/CRC group. Connecting dotted line is placed above bars that represent statistically different means between the control and CRC groups on the same feed at *p* < 0.05.

Additional analysis was performed to confirm the presence of chosen specific metabolites, selected from a list of metabolites that differed significantly between the CRC group and other experimental groups included in the metabolomic analysis (CON, CRC_BG_1, CRC_BG_3). After the initial verification of metabolites using the ChemSpider platform, a LC-MS/MS based targeted metabolomic analysis was performed with a thorough analysis of the fragmentation spectra. Only the confirmation of these spectra ensured that the metabolites were correctly selected for further research. Out of the initially selected 12 metabolites, 4 were conclusively confirmed: kynurenine, methionine, citrulline and tryptophan. These compounds showed significantly higher levels in the CRC group in comparison to the control (CON) groups and CRC groups fed the diet supplemented with OBG, regardless of the dose used (CRC_BG_1 and CRC_BG_3) (Figure 5). The consumption of the feed supplemented with OBG led to a reduction in the levels of kynurenine, methionine, citrulline, and tryptophan, bringing them in line with the values observed in the colorectal samples from rats in the control groups (Figure 5). The role of these compounds is described in more detail in the discussion section.

## 3. Discussion

The presented study aimed to determine the effect of low-molar-mass oat beta-glucan (OBG) on oxidative stress parameters, inflammation, and the metabolomic profile of the large intestine tissue in rats exposed to azoxymethane. The AOM model is a well-documented and widely used method for experimentally induced colorectal carcinogenesis [12]. This model, known for its resemblance to sporadic colorectal cancer in humans, not only shares similarities in response to certain promotional and preventive factors but also serves as an effective tool in evaluating the chemopreventive mechanisms of action of biologically active compounds in this specific type of cancer [13,14].

There are many types of beta-glucans, and the structure of these polysaccharides differs depending on the source of origin. Fungal beta-glucans are polymers of D-glucose residues joined in a β-1,3 configuration with additional β-(1,6)-linked branches that show a strong immunomodulatory effect [15]. In turn, branched or linear 1,4-β-glucans are believed to show limited biological activity [15]. Our research investigated the effect of OBG administered to rats for 8 weeks in the form of a feed supplement. The tested beta-glucan was isolated from oat bran and purified by a patented method described in detail elsewhere [16,17]. The structure of OBG is characterized by a mixture of β-D-glucose unbranched chains linked by β-(1,3) and β-(1,4) glycosidic bonds. Our previous reports demonstrated that the examined low-molar-mass OBG administered at a dose of 1% feed additive had a strong direct anti-inflammatory effect as well as an indirect antioxidant effect in models of colitis induced by 2,4,6-trinitrobenzenesulfonic acid (TNBS) and systemic inflammation induced by lipopolysaccharides (LPS) in rats [6,18,19]. To test whether the effectiveness of OBG is maintained or modified by an increased dose of OBG present in the diet, we also investigated the effect of 3% OBG.

One of the objectives of the present study was to investigate the antioxidative effect of dietary supplementation with OBG in animals with AOM-induced early-stage CRC. The role of oxidative stress in cancer, characterized by the overproduction of reactive oxygen species (ROS) and reactive nitrogen species (RNS) with simultaneous deficiency in endogenous antioxidant systems, has been extensively explored in many studies focused on carcinogenesis in the digestive tract, including the colon. Endogenous antioxidative systems include mainly superoxide dismutase (SOD), glutathione in reduced and oxidized forms, enzymes involved in glutathione metabolism (glutathione peroxidase and reductase) and others. Inhibition of the key enzymes involved in the synthesis of glutathione or enzymes capturing ROS causes prolonged oxidative stress, which may lead to induction and intensification of the process of carcinogenesis [20,21]. On the other hand, the inhibitory effect of biologically active compounds on redox processes in cancer cells suggests the possibility of their chemopreventive properties. In our study, AOM-induced CRC caused a statistically significant decrease in the GSH concentration (glutathione in reduced form), which also resulted in a decrease in the value of the GSH/GSSG ratio. Furthermore, we found increased activity of glutathione peroxidase (GPx) and decreased activity of glutathione reductase (GR) in all groups of animals with induced early-stage CRC. The 8-week dietary intervention with OBG, regardless of its dose (1% or 3%), improved the parameters characteristic for antioxidant protection related to glutathione oxidation status. Although the low-molar-mass OBG did not fully restore the activity of GPx and GR to the values observed in control groups, it modulated those changes towards physiological levels, particularly by decreasing GPx activity and increasing the activity of GR. The effect was especially pronounced in the case of 1% OBG supplementation. The mechanism of action of the low-molar-mass OBG in our experimental model does not seem to involve direct inhibition of AOM-induced carcinogenesis because no available data confirm the direct absorption of the beta-glucan into the intestinal cells. Oat beta-glucan exhibits high solubility in water and forms viscous gels due to long, poorly branched polysaccharide chains linked mainly by β-1,4-glycosidic bonds. The probable antioxidative mechanism of OBG action is based on limiting the promotion of the secondary oxidative factors that lead to the generation of oxidative stress. This causes the decreased concentration of free radicals, and thus, the diminished oxidation of GSH.

Glutathione peroxidase can also prevent the formation of toxic lipid peroxides by reducing ROS concentration in the cells [22]. Thus, we also analysed the concentration of lipid peroxidation products using the TBARS assay. The products of lipid peroxidation are used as markers of the degree of lipid damage, and the formed lipid peroxides show pro-mutagenic properties and induce mutations in oncogene/tumour suppressor genes in human cancers as well as in animal research models [23,24]. In our study, the colorectal tissue of rats with AOM-induced CRC showed a strong accumulation of lipid peroxidation products, expressed as the sum of TBARS. The lowest TBARS concentration was noted in control groups, irrespective of OBG supplementation, indicating that the protective effect of OBG was not observed in healthy animals.

Among the crucial parameters determining the ability of cells to resist oxidative stress is also superoxide dismutase (SOD). In the cancer cells microenvironment, there are several metabolites that activate enzymes involved in elimination of the effects of oxidative stress due to their pro-oxidative nature. The increase in SOD activity has been correlated with the increase in colorectal cancer severity, which has been demonstrated in neoplastic tissues compared to normal tissues [25]. The results from other studies have shown increased SOD activity in colon samples from patients with colon cancer at stages I and III when compared to the control group, whereas the lower SOD activity was demonstrated in patients with colon cancer at stage IV [26,27]. In our study, the SOD activity of animals in the control group (CON) was lower than that in the CRC group, confirming the initial stages of CRC development. This was also reflected by the results of the histopathological examination of colon sections from CRC and CON groups, revealing the initial stages of crypt aberration under the influence of AOM treatment, without noticeable changes in inflammatory cells infiltration. The consumption of feed supplemented with OBG, regardless of its concentration, significantly reduced SOD activity, confirming the potent strong chemopreventive effect of these polysaccharides in animals with early-stage CRC development. Taking together the results of the redox status of the colorectal tissue (GSH and GSSG concentrations, activity of GSH-related enzymes, activity of SOD, and TBARS concentration), it can be concluded that low-molar-mass OBG exhibits the potential to modulate the antioxidative balance in tissues altered by the early stage of CRC development, significantly diminishing the induced oxidative stress in the cancerous environment, but the effect did not depend on the doses selected in the present research.

In order to create a favourable tumour microenvironment, cancer cells primarily communicate with each other mainly through signalling molecules, such as cytokines, chemokines, or growth factors [28]. Nutritional interventions aim at modifying the secretory profile of the pro- and anti-inflammatory cytokines in order to minimize the risk of cancer development, growth, invasion, metastases, and resistance to therapy. Dietary bioactive compounds, such as beta-glucans, most probably are unable to reach further organs in an unchanged form. Their mechanism of action is limited to specific sections of the digestive tract, especially the large intestine. It is believed that oat beta glucans stimulate the immune cells to produce anti-inflammatory cytokines and inhibit the secretion of pro-inflammatory cytokines [29,30]. Our present study demonstrated that the concentration of pro-inflammatory interleukins IL-1α, IL-1β, IL-12, and C-reactive protein (CRP) was increased in the colorectal tissues of rats with AOM-induced CRC, whereas the concentration of the anti-inflammatory IL-10 was decreased in CRC. Supplementation of feed with the low-molar-mass OBG led to decreased concentrations of the pro-inflammatory cytokines (IL-1α, IL-1β, IL-12) in the colorectal tissue of rats with AOM-induced CRC to levels comparable with healthy control rats. Our previous research also showed that dietary supplementation with oat beta-glucans protected the large intestine from inflammation, in that case induced by LPS [6]. Since chronic inflammation may lead to epithelial–mesenchymal transition, dedifferentiation, and increased ROS production in cancer cells [31], our results support the hypothesis of the anti-cancer activity of the oat beta glucans, reflected among others by their potential for regulating the antioxidative balance and anti-inflammatory response.

One of the major objectives of the present study was to assess the difference in the metabolomic profile of colorectal tissue under physiological state (CON) and pathological conditions (CRC) induced by the AOM treatment. Additionally, the study aimed to identify metabolites that may exhibit differences after dietary intervention with the low-molar-mass oat beta-glucan. Therefore, for this purpose, metabolome comparisons of CRC vs. CON, CRC vs. CRC_1BG, and CRC vs. CRC_3BG were performed separately.

As a result of early-stage colorectal cancer induction by AOM treatment, changes in several key metabolic pathways were demonstrated. Some of the activated metabolic pathways have been previously described in the literature. Recently, a systemic review of studies reported from January 2012 to July 2021 described CRC biomarkers detected by metabolomics, pointing out metabolites that may be used as potential diagnostic markers in the future [32]. However, there are no data characterizing the metabolic profile of the large intestine under physiological conditions. Therefore, we compared the results of metabolomic analysis performed on control colorectal tissue samples with the CRC samples. Among the metabolic pathways that differed between the compared experimental conditions (CRC vs. CON), the crotonoyl-CoA and butanoyl-CoA pathway, involved in the beta-alanine pathway, biotin metabolism, butanoate and folate metabolic pathways, purine metabolism, and one-carbon metabolism, mediated by the folate cofactor, may be of special interest. Folate, acting as a donor and carrier of one-carbon units, is involved in various processes within the one-carbon metabolism, including the biosynthesis of guanine and adenine nucleotides necessary for nucleic acid synthesis [33]. In our study the pathways of purine metabolism were among the pathways differentially regulated in CRC group when compared with control group (CON). Moreover, carcinogenesis induction by AOM also induced changes in the sphingolipid pathway and a large part of amino acids metabolism. Sphingolipid metabolites can mediate several signalling pathways, as well as affect cell growth, differentiation, autophagy, and apoptosis [34].

Our study also revealed a number of metabolic pathways regulated by the low-molar-mass OBG administered as a dietary supplement at 1% or 3% concentration. It seems that the most important metabolic pathways altered by 1% OBG are involved in the metabolism of polyunsaturated fatty acids (PUFAs), specifically arachidonic acid, linoleic acid, and their metabolites, as well as the metabolism of glycerophospholipids. Arachidonic acid metabolites are crucial for the function of cell membranes of cells stimulated by onco-carcinogens. Gholamalizadeh et al. [35] showed a relationship between the activity of enzymes involved in arachidonic acid metabolism and the type of fat in the diet, with highly inflammatory eicosanoids being products of those enzymatic reactions. Our study confirmed that the dietary oat beta-glucan reduced the concentration of pro-inflammatory cytokines, and given that PUFAs are precursors of prostanoids involved in inflammation, the modulation of arachidonic acid metabolism becomes particularly relevant. Prostanoids, which include leukotrienes, thromboxanes, and prostaglandins, are powerful signalling molecules synthesized by a diverse set of enzymes during inflammation. Among them, prostaglandin E2 (PGE2), the main product of cyclooxygenase (COX), plays a significant role in colorectal carcinogenesis. Many studies showed elevated levels of PGE2 in colon cancer [36]. An analysis using MetaboloAnalyst suggested that the change in the entire metabolic pathway of arachidonic acid was influenced to the greatest extent by the presence of the eicosanoids 16(R)-HETE, 9(S)-HETE, 11.12-EET, 5(S)-HETE, Hepoxilin A3-C, 15(S)-HETE, 19(S)-HETE, and 20(S)-HETE, which, as isomers, would require a thorough analysis in order to confirm their real impact on the metabolome of the tested samples. Despite the attempts of chromatographic separation and their analysis, we were unable to confirm the presence of these eicosanoids quantitatively. However, taking into account the significant changes in the arachidonic acid metabolic pathway and our previous observations regarding the decreased concentration of PGE2 in the colon tissue of rats with TNBS-induced colitis, receiving low-molar-mass OBG [19], it can be concluded that oat beta-glucans play an important role in the regulation of arachidonic acid metabolism.

The addition of OBG to the feed of rats with AOM-induced CRC, especially at the higher concentration (3%), also increased the concentration of metabolites involved in the pathway of methionine and cysteine metabolism. Cysteine, in particular, plays a key role in regulating the tumour microenvironment. This sulphur-containing amino acid takes part in cell biosynthesis processes, in enzymatic catalysis and, above all, in redox metabolism. Extracellular cysteine serves as the primary source for the intracellular cysteine pool that supports the cellular redox state [37]. During carcinogenesis, the demand for cysteine increases for glutathione production to manage oxidative stress. In our research, an increase in the GSH/GSSG ratio was noted in tissue samples from rats with AOM-induced CRC fed a diet supplemented with OBG (CRC_BG_1, CRC_BG_3) in comparison to colorectal tissue samples from the CRC group. These finding confirm the antioxidative properties of OBG, as discussed earlier, and show the relationship between the results of our metabolomic analysis with the investigated oxidative status.

Methionine, the second sulphur-containing amino acid, initiates protein synthesis during translation and serves as a source of methyl groups for most nucleotides, chromatin, and protein methylation. It also donates carbon skeletons for various aspects of the cellular antioxidant response and nucleotide biosynthesis. Cancer cells, unlike normal cells, exhibit high methionine cycle activity and depend on exogenous methionine for continued growth [38]. Methionine deficiency initiates extensive metabolic changes in cancer cells that enable them to survive despite the limited availability of this amino acid [39]. Furthermore, the combined effects of methionine and cysteine are important because of their direct involvement in the metabolism of the glutathione pathways and the antioxidant capacity of cells.

Our investigation also revealed the altered metabolism of glycine, serine, alanine, and threonine in the colorectal tissue of rats treated with AOM and fed a diet containing 3% of OBG. Glycine, as a source of one-carbon units, together with increased expression of glycine decarboxylase, promotes the growth of cancer cells, e.g., for colorectal cancer [40]. Interestingly, an excess of glycine can be harmful to cancer cells, leading to a reduction in the rate of proliferation and inhibiting tumour growth. In colorectal cancer cells with an excess of glycine and a serine deficiency, the dominant reaction catalysed by methyltransferases is the conversion of glycine to serine. This reaction not only results in the depletion of one-carbon units but also inhibits nucleotide synthesis [41]. Studies have shown that the relationship between the breakdown of serine and the formation of glycine and the direction of these changes is crucial for colon cancer cell survival [41]. The observed changes in amino acid metabolism in our study emphasize the potential influence of OBG on the one-carbon metabolism and nucleotide synthesis in CRC.

Finally, four metabolites (kynurenine, methionine, citrulline, and tryptophan) were selected in the present study for further quantitative and qualitative analysis, thus allowing us to confirm the changes in their levels in the investigated experimental groups. Kynurenine plays a vital role in the nutritional modulation of immune cells and promotes the differentiation of Treg lymphocytes, which leads to the increased production of anti-inflammatory cytokines and inhibition of cytotoxic activity of T lymphocytes. However, an excessive activation of the kynurenine pathway in tumours has been linked to increased survival and invasion of tumour cells into surrounding tissues [42]. The kynurenine pathway strongly interacts with other molecular pathways involved in tumorigenesis, including the Wnt/β-catenin pathway, which is directly related to the AOM mechanism of action, and the COX2 and cyclin-dependent kinase pathways. The over-activation of the kynurenine pathway predicts a poor prognosis for several cancers, such as gastrointestinal cancers [43]. In our study, the quantitative analysis confirmed that in the group of rats with CRC and consuming feed supplemented with the oat beta-glucan (CRC_BG_1 and CRC_BG_3), the concentration of kynurenine decreased to the concentrations observed in animals from the control group (CON), while the highest concentration was observed in the large intestine of animals from the CRC group. Such modulation may contribute to a less favourable environment for tumour survival and invasion.

It is also important to link the metabolism of kynurenine with tryptophan (TRP)—one of the essential, exogenous amino acids, which plays an important role in both nutrition and a variety of physiological processes in organisms [44]. Two main metabolic pathways for tryptophan are known in mammals: the kynurenine metabolism pathway and the serotonin metabolism pathway. The former plays a significant role in the immune function and, therefore, in development [45]. Tryptophan is converted to kynurenine through the actions of enzymes such as tryptophan 2,3-dioxygenase (TDO) or indoleamine 2,3-dioxygenase (IDO). In the gastrointestinal tract and brain, residual TRP is transformed into kynurenine via IDO. Kynurenine then undergoes further metabolism leading to the production of various metabolites, including kynurenic acid (KA) and quinolinic acid, which is later transformed to NAD^+^. Additionally, the gut microbiome can metabolize TRP to indole and its derivatives [46]. Tryptophan and its metabolites have multiple important functions in the gastrointestinal tract. Firstly, metabolites of tryptophan are believed to be ligands for the aryl hydrocarbon receptor (AhR), which is responsible for modulating immune response in the intestine [44]. Studies suggest that the number of T cells can be positively influenced by the high intake of tryptophan from the diet, and the indole acrylic acid improves the overall function of the epithelial barrier in the intestine [47,48]. Furthermore, TRP as well as kynurenine can inhibit the inflammatory response [49]. In the studies on CRC progression, the kynurenine pathway has been connected with an inhibitory effect on CRC cell proliferation. In the present research, the tryptophan level was reduced to control physiological values in the colorectal tissue of CRC rats fed the diet supplemented with OBG regardless of the dose used. This suggests a potential influence of oat beta-glucan on tryptophan metabolism and its associated pathways. The modulation of tryptophan metabolism, especially within the kynurenine pathway, appears to be a critical factor in the complex interplay between diet, immunity, and colorectal cancer. Understanding these intricate mechanisms provides valuable insights for potential therapeutic interventions and emphasizes the importance of dietary components in influencing metabolic responses.

## 4. Materials and Methods

### 4.1. Isolation and Characteristics of OBG

Beta-glucan was extracted from oat bran concentrate (Bestpharma, Warsaw, Poland) using a unique method described in details in our previous reports [16,17]. The detailed methodology for the isolation and purification of beta-glucan was described in our previous article [50]. Briefly, the oat bran was frozen and subjected to repeated milling, and was then extracted using an alkaline solution. The remaining substances were then separated by centrifuging and the supernatant was de-proteinised at its isoelectric point. The protein content, measured using the Lowry method, was found to be negligible, falling below the method’s detection limit (<0.01 mg/mL). The purity of the beta-glucan was 99.3%, as determined by enzymatic methods (AACC Method 32-23.01). The molar mass of the beta-glucan used in the present study was determined by SEC-HPLC to be approximately 5.2 × 10^4^ ± 0.6 × 10^4^ g/mol.

### 4.2. In Vivo Experiment

The experiment was conducted on eight-week old male Sprague Dawley rats (CRL:CD(SD)), purchased from Charles River Laboratories (Sulzfeld, Germany). Detailed description of the in vitro experiment was presented in our recent research article [50]. Briefly, after a one-week adaptation period, the animals were divided into 2 main groups: a treatment group with chemically induced early-stage colorectal cancer (CRC, n = 24), and a control group, without pathological colorectum changes (CON, n = 21). The CRC was induced by peritoneal injection of azoxymethane (AOM) (Sigma-Aldrich, Saint Louis, MO, USA) at a dose of 15 mg/kg once a week for two weeks in accordance with the method described by Perše and Cerar [7]. Animals in control group (CON) received peritoneal injections of physiological saline using the same scheme of treatment. On the day of last AOM injection, both CRC and CON groups were subdivided into three sub-groups: (1) animals fed AIN-93M feed (ZooLab, Sędziszów, Poland) without beta-glucans (groups CRC and CON), (2) animals fed AIN-93M feed supplemented with 1% (*w*/*w*) of OBG (groups CRC_BG_1 and CON_BG_1), and (3) animals fed AIN-93M feed supplemented with 3% OBG (*w*/*w*) (groups CRC_BG_3 and CON_BG_3). Each CRC subgroup comprised 8 animals, whereas each CON subgroup comprised 7 animals. Detailed description of feed composition in all experimental groups and environmental conditions were provided in our previous article [50]. Each animal’s body weight was recorded weekly, and feed consumption was monitored every day. At the end of the 56-day feeding period, the animals were subjected to general Isoflurane anaesthesia and blood was sampled by heart puncture, and after bleeding, the three parts of large intestine (cecum, colon, and rectum) were sampled. In order to minimize the impact of the ingesta components, all sampled parts were thoroughly rinsed in phosphate-buffered saline (PBS), then frozen in liquid nitrogen and stored at −80 °C for further analysis.

All experimental procedures were approved by the 2nd Local Ethical Committee in Warsaw, Poland (resolution no. WAW2/040/2019), in accordance with the EU Directive (2010/63/UE), the Polish law, and the 3R rules.

### 4.3. Histopathological Evaluation

Samples of the large intestine were fixed in 10% buffered formaldehyde, and dehydrated by successive immersion in a gradient of ethanol solutions. Subsequently, the samples were rinsed with xylene, embedded in paraffin blocks, and cut into 5 μm sections. The sections were stained with haematoxylin and eosin (H&E) for evaluation under a light microscope (Motic BA400, Olympus Corporation, Tokyo, Japan) by a veterinarian histopathologist.

### 4.4. The Immunological Parameters Evaluation

The large intestine samples were homogenized in PBS using electric homogenizer, vortexed for 15 min and centrifuged for 15 min at 500× *g*. Supernatants were used to analyse the concentrations of chosen cytokines interleukin IL-10 (cat.no.: RBMS629R), IL-12 (cat.no.: E90059Ra), IL-1α (cat.no.: E900233Ra), IL-1β (cat.no.: E900235Ra), and C reactive protein (CRP, cat.no.: E900878Ra) by competitive specific enzyme immunoassay (ELISA) commercial kits (USCN Life Science Inc., Wuhan, China). Analyses were performed according to the manufacturer’s protocols.

### 4.5. The Colon Redox Balance Parameters Evaluation

Determination of total antioxidant status (TAS), glutathione reductase (GR), glutathione peroxidase (GPx), and superoxide dismutase (SOD) activity was conducted in tissue homogenates prepared according to the protocol described in Section 4.4. The parameters were analysed using the Randox assay kits (TAS, cat. no.: NX2332; GR, cat. no.: GR 2368; GPx, cat. no.: RS504/505/506; SOD, cat. no.: SD125) according to the protocols provided by the producer (Randox Laboratories Ltd., Crumlin, County Antrim, UK). Reduced (GSH) and oxidized (GSSG) gluthatione were detected using high-pressure liquid chromatography (HPLC) with a colourimetric electrochemical detector from ESA (Chelmsford, MA, USA) with a 4-channel electrochemical array for the simultaneous detection of both glutathione forms. The mobile phase for isocratic elution of the sulfhydryls was composed of 25 mM monobasic sodium phosphate, 0.5 mM 1-octane sulfonic acid, and 2.5% acetonitrile, adjusted to pH 2.7. All chemicals, including GSH and GSSG standards were purchased from Sigma-Aldrich (part of Merck KGaA, Darmstadt, Germany). The pH for the mobile phase was adjusted with 85% phosphoric acid. A flow rate of 1 mL min^−1^ was used with a C18 column (5 μM column, 4.6 × 250 mm). Acetonitrile was the key component in modulating the retention times of GSH and GSSG. With 2.5% of acetonitrile in the mobile phase, the retention times for GSH generally appeared at 5 min and GSSG at 20 min. The GSH/GSSG ratio was calculated on this basis. Thiobarbituric acid reactive substances (TBARSs) were analysed according to the method described by Aguilar Diaz De Leon and Borges [51].

### 4.6. Metabolomic Analysis

Metabolomic analysis was performed using a high-pressure liquid chromatography Symbiosis Pico UHPLC system. The detector used for this analysis was a SCIEX TripleTOF 5600+ DuoSpray Source for SCIEX TripleTOF 5600+ (TurboIonSpray and APCI). The acquired data were analysed using SCIEX MarkerView™ (ver. 1.3.1.), XCMSplus (on-line ver.), and MetaboAnalyst 5.0 software packages for comprehensive interpretation and extraction of metabolomic information.

The samples of colon tissue were homogenized in PBS and mixed with a mixture of acetonitrile and methanol in a 1:1 ratio. Samples were vortexed (2000 rpm for 15 min) and centrifuged for 15 min at 20,000× *g*. Supernatants were transferred to glass autosampler vials, and placed in an autosampler at 4 °C. Samples were injected directly into a Spark Holland Symbiosis™ Pico system. Chromatographic separation was performed on the Hypersil chromatographic column, BDS C18, (150 × 4.6 mm, 5 μm) with a Hypersil C18 guard column (10 × 2.1 mm, size 5 μm). The mobile phase consisted of methanol/formic acid (99:1, *v*/*v*) A and water/formic acid (99:1, *v*/*v*) B, and the flow rate was constant at 500 µL min^−1^. The gradient elution of the mobile phase 100% A was started at 1.1–40 min linear gradient to 100% B, 40.1–55 min 100% B, and 55.1–60 min linear gradient to 100% A.

MS/MS parameters: the optimized detection conditions included curtain gas (N_2_) 25 psi, nebulizer gas (N_2_) 20 psi, heater gas (N_2_) 50 psi, ion source voltage floating 5500 V, and source temperature 500 °C. Samples with a heated electrospray 3 ionization probe were measured in positive ionization (H-ESI+).

### 4.7. Statistical Analyses

Analyses of variance (ANOVA) were performed using GraphPad Prism ver.6.0 (GraphPad Software, Inc., La Jolla, CA, USA). To determine the effect of AOM treatment, as well as supplementation of the diet with 1% or 3% of OBG, two-way ANOVA was employed to assess differences between control and experimental groups. The significance of differences in results among the groups was determined by Tukey’s post hoc test. A significance level of *p* < 0.05 was considered as a statistically significant difference. All data were expressed as mean ± standard deviation (mean ± SD).

The metabolite profiles, obtained within the 100–1100 Da range with a sensitivity of 5 cps, were analysed using SCIEX MarkerView™ software (ver.1.3.1.). Comparative assessments between groups were conducted using Student’s *t*-tests and principal component analysis (PCA). The generated metabolomic profiling data sets were processed by the control software of the SCIEX Analyst^®^ (ver.1.7.3) mass spectrometer and saved in a specific data format (*.raw). The first step was to convert data from Excalibur-specific raw files to open format files (*.mzXML) using MS Convertor software (MSConvert ver. 1.5.2.). Subsequently, the metabolomic data were processed using the XCMSplus platform. Additionally, PCA resulted in comparative profiles of metabolomes of specific groups, which were further analysed by SCIEX MarkerView™ software. The classification of identified metabolites within particular metabolic pathways was conducted using the XCMSplus on-line platform and MetaboAnalyst 5.0 software with a probability threshold of *p* < 0.0001. The identification of metabolites was carried out based on the ChemSpider database (accessed via SCIEX PeakView™ ver.2.2). Indications above an 80% probability of confirming a given structure were compared with the indications of metabolites from the MetaboAnalyst 5.0 database.

## 5. Conclusions

The obtained results indicate that dietary supplementation with the low-molar-mass oat beta glucan may play an important role in maintaining homeostasis of the intestinal microenvironment. The observed effects include exerting the anti-inflammatory and antioxidant activities, emphasizing the potential use of oat beta-glucan in dietary intervention. Additionally, low-molar-mass oat beta-glucan was found to modulate metabolic pathways, specifically those related to amino acids and fatty acids metabolism, which are crucial for maintaining the physiological functions of the colorectal tissue. The presented study is consistent with contemporary trends of research investigating the role of biologically active compounds in the prevention of colon cancer. However, only one type of beta-glucans was investigated, which does not allow for a direct comparison of the mechanism of action of beta-glucans of different origin and structure. Nevertheless, the present research, which refers only to oat beta-glucans, documents their strong effect in the early stages of carcinogenesis. Our results suggest potential therapeutic implications for dietary oat beta-glucan interventions aimed at supporting the intestinal homeostasis. The potential therapeutic use of beta-glucans isolated from oat grains should be further explored in the future preclinical and clinical studies.

## Figures and Tables

**Table 1:** Concentration of reduced (GSH) and oxidized glutathione (GSSG), GSH/GSSG ratio, activity of glutathione reductase (GR) and glutathione peroxidase (GPx) in colorectal tissue of rats from control (CON) and AOM-induced colorectal cancer (CRC) groups fed a diet with 1% or 3% addition of oat beta-glucan (BG_1 and BG_3, respectively) or control diet.

Parameters	CON Group			CRC Group		
	CON (*n* = 7)	CON_BG_1 (*n* = 7)	CON_BG_3 (*n* = 7)	CRC (*n* = 8)	CRC_BG_1 (*n* = 8)	CRC_BG_3 (*n* = 8)
GSH	269.89 ± 11.2 ^a,^*	223.72 ± 12.6 ^b,^*	230.26 ± 18.5 ^b,^*	109.05 ± 13.3 ^a,^*	165.82 ± 13.4 ^b,^*	172.99 ± 17.6 ^b,^*
GSSG	346.5 ± 25.7 *	357.5 ± 12.1 *	314.3 ± 17.6 *	712.9 ± 72.9 ^a,^*	473.4 ± 47.0 ^ab,^*	588.5 ± 73.4 ^a,^*
GSH/GSSG	0.68 ± 0.07 ^a,^*	0.56 ± 0.03 ^b^*	0.67 ± 0.08 ^a,^*	0.13 ± 0.02 ^a,^*	0.31 ± 0.05 ^b,^*	0.31 ± 0.07 ^b,^*
GR	2.60 ± 0.22 *	2.55 ± 0.07 *	2.521 ± 0.10 *	1.71 ± 0.14 ^a,^*	2.07 ± 0.17 ^b,^*	1.78 ± 0.15 ^ab,^*
GPx	150.85 ± 11.5 *	175.59 ± 6.8 *	170.97 ± 10.9 *	331.22 ± 30.1 ^a,^*	246.43 ± 12.4 ^b,^*	296.63 ± 25.2 ^a,^*

All values were expressed as means ± standard deviation. Statistically significant differences between groups and within groups were evaluated using two-way ANOVA with Tukey’s post hoc test; ^a,b^—different letters denote significant differences among dietary subgroups in the control/CRC group at *p* < 0.05; *—denotes significantly different results between the control and CRC groups on the same feed at *p* < 0.05. GSH and GSSG concentration is expressed in μmol/g of wet tissue; GR and GPx activity is expressed in U/g of wet tissue.

## Data Availability

The data that support the findings of this study are available on request from the corresponding author.

## Supplementary Materials

The following supporting information can be downloaded at: https://www.mdpi.com/article/10.3390/ijms25094635/s1.
