# Supplementary material for: Oat Beta-Glucan as a Metabolic Regulator in Early Stage of Colorectal Cancer—A Model Study on Azoxymethane-Treated Rats
Source: Int J Mol Sci. 2024 Apr 24;25(9):4635. doi: 10.3390/ijms25094635 (PMC11083532; doi:10.3390/ijms25094635)
Supplement: Supplementary file 1 [file ijms-25-04635-s001.zip › ijms-2934678-supplementary.pdf]

Table S1a. The list of differentiated metabolic pathways in colon tissue of rats from **CRC** and **CON** groups.

Pathway Name	Total	Hits (all)	Hits (sig.)	P-value	Gamma P
Butanoate metabolism	15	3	2	0.00022	0.02065
Valine, leucine and isoleucine degradation	35	13	3	0.00685	0.02292
Fatty acid degradation	36	14	3	0.09971	0.02652
Steroid hormone biosynthesis	77	22	2	0.18582	0.03761
Pentose phosphate pathway	21	4	1	0.2474	0.04288
Fatty acid elongation	30	11	1	0.24547	0.04568
Purine metabolism	66	5	1	0.42173	0.07146
Lysine degradation	19	2	1	0.32974	0.07541
Tryptophan metabolism	41	2	1	0.61451	0.09207
beta-Alanine metabolism	21	4	1	0.45051	0.09222
Sphingolipid metabolism	9	2	1	0.45051	0.09222
Propanoate metabolism	19	4	1	0.55791	0.10957
One carbon pool by folate	9	6	1	0.55791	0.10957
Riboflavin metabolism	4	3	1	0.82401	0.11407
Biotin metabolism	4	1	1	0.7283	0.11545
Folate biosynthesis	24	4	1	0.77702	0.13848
Retinol metabolism	15	5	1	0.72546	0.14491

Table S1b. The list of significantly ($p<0.05$) regulated metabolites in colon tissue of rats from **CRC** and **CON** groups.

**Name**	**Pathway Name**	**Exact mass**	**P value**	**FC**	**logFC**	**Remarks**
3-Methylcrotonyl-CoA	Butanoate metabolism	849.1571	0,01377	4,56	1,76	
pentadecanoylcarnitine	Valine, leucine and isoleucine degradation	715,5012	0,0001	3,71		Metabolite absent in CRC
Dehydroepiandrosterone sulfate	Fatty acid degradation	284,4352	2,54E-05	3,66	0,56	
13,14-Dihydroxy-Retinol	Steroid hormone biosynthesis	313,4533	2,54E-05	3,65	1,65	
Thiopurine	Pentose phosphate pathway	152,1772	2,54E-05	3,55		Metabolite absent in CRC
Coproporphyrin I	Fatty acid elongation	197,1032	0,00138	3,49	0,55	
Coproporphyrin III	Purine metabolism	300,4352	0,00138	3,38		Metabolite absent in CRC

S-(11-OH-9-deoxy-delta9,12-PGD2)-glutathione	Lysine degradation	398,6643	7,24E-08	3,34	0,77	
S-(9-deoxy-delta12-PGD2)-glutathione	Tryptophan metabolism	654,7083	0,00538	3,33	0,54	
10,11-dihydro-12R-hydroxy-LTC4	beta-Alanine metabolism	336,4652	1,59E-15	3,23		Metabolite absent in CRC
Dehydroepiandrosterone sulfate	Sphingolipid metabolism	182,1732	0,00057	3,02	0,49	
L-Fucose 1-phosphate	Propanoate metabolism	244,1361	0,00057	3,01	0,48	

Table S2a. The list of differentiated metabolic pathways in colon tissue of rats from **CRC** and **CRC_BG_1** groups.

Pathway Name	Total	Hits (all)	Hits (sig.)	P-value	Gamma P
Arachidonic acid metabolism	95	26	2	0.85296	0.11185
Linoleate metabolism	46	10	1	0.72658	1.0
Carnitine shuttle	72	1	1	0.11834	1.0
Androgen and estrogen biosynthesis and metabolism	95	22	1	0.94885	1.0
Vitamin A (retinol) metabolism	67	10	1	0.72658	1.0
Glycerophospholipid metabolism	156	6	1	0.53602	1.0
Porphyrin metabolism	43	9	1	0.68752	1.0
Prostaglandin formation from arachidonate	78	9	1	0.68752	1.0
Leukotriene metabolism	92	9	1	0.68752	1.0
C21-steroid hormone biosynthesis and metabolism	112	17	1	0.89537	1.0

Table S2b. The list of significantly (p<0.05) regulated metabolites in colon tissue of rats from **CRC** and **CRC_BG_1** groups.

**Name**	**Pathway Name**	**Exact mass**	**P value**	**FC**	**logFC**	**Remarks**
16(R)-HETE; 9(S)-HETE; 11,12-EET; 5(S)-HETE; 19(S)-HETE	Arachidonic acid metabolism	320.2351	0,01409	57,70	1,76	
pentadecanoylcarnitine	Carnitine shuttle	385.6232	0,00003	3,65		Metabolite absent in CRC_3BG
Dehydroepiandrosterone sulfate	Androgen and estrogen	368.1657	0,00138	3,60	0,56	

	biosynthesis and metabolism					
13,14-Dihydroxy-Retinol	Vitamin A (retinol) metabolism		0,00138	3,60	0,56	
20-HETE	Glycerophospholipid metabolism	320.2351	0,00000	3,54		Metabolite absent in CRC_3BG
Coproporphyrin I	Porphyrin metabolism	654.269	0,00538	3,51	0,54	
Coproporphyrin III		654.269	0,00000	3,33		Metabolite absent in CRC_3BG
S-(11-OH-9-deoxy-delta9,12-PGD2)-glutathione	Prostaglandin formation from arachidonate		0,00057	3,02	0,48	
S-(9-deoxy-delta12-PGD2)-glutathione			0,00057	3,02	0,48	
10,11-dihydro-12R-hydroxy-LTC4	Leukotriene metabolism		0,00011	2,78	0,44	
Dehydroepiandrosterone sulfate	C21-steroid hormone biosynthesis and metabolism	368.1657	0,00011	2,78	0,44	

Table S3a. The list of differentiated metabolic pathways in colon tissue of rats from **CRC** and **CRC_BG_3** groups.

Pathway Name	Total	Hits (all)	Hits (sig.)	P-value	Gamma P
Aspartate and asparagine metabolism	114	3	2	0.15282	0.01631
Methionine and cysteine metabolism	94	3	2	0.15282	0.01631
Di-unsaturated fatty acid beta-oxidation	26	4	2	0.25783	0.023761
Mono-unsaturated fatty acid beta-oxidation	19	4	2	0.25783	0.023761
Leukotriene metabolism	92	9	3	0.39493	0.025033
Omega-6 fatty acid metabolism	55	5	2	0.36394	0.032614
Fatty acid activation	74	6	2	0.46424	0.04268
De novo fatty acid biosynthesis	106	7	2	0.55508	0.053752
Porphyrin metabolism	43	8	2	0.6349	0.065621
Prostaglandin formation from arachidonate	78	9	2	0.70346	0.078104
Arachidonic acid metabolism	95	26	4	0.93431	0.13341
C21-steroid hormone biosynthesis and metabolism	112	17	2	0.95686	0.18603
Androgen and estrogen biosynthesis and metabolism	95	22	2	0.98914	0.25293
Vitamin D3 (cholecalciferol) metabolism	16	1	1	0.24848	1.0
Glycolysis and Gluconeogenesis	49	1	1	0.24848	1.0
C5-Branched dibasic acid metabolism	10	1	1	0.24848	1.0

Pyruvate Metabolism	20	1	1	0.24848	1.0
Linoleate metabolism	46	10	1	0.94772	1.0
Squalene and cholesterol biosynthesis	55	2	1	0.43636	1.0
3-oxo-10R-octadecatrienoate beta-oxidation	27	1	1	0.24848	1.0
Vitamin B1 (thiamin) metabolism	20	1	1	0.24848	1.0
Histidine metabolism	33	2	1	0.43636	1.0
Propanoate metabolism	31	1	1	0.24848	1.0
Vitamin E metabolism	54	4	1	0.6849	1.0
Glycine, serine, alanine and threonine metabolism	88	2	1	0.43636	1.0
Drug metabolism - cytochrome P450	53	1	1	0.24848	1.0
Tryptophan metabolism	94	1	1	0.24848	1.0
Urea cycle/amino group metabolism	85	1	1	0.24848	1.0
Valine, leucine and isoleucine degradation	65	3	1	0.57814	1.0

Table S3b. The list of significantly ($p<0.05$) regulated metabolites in colon tissue of rats from **CRC** and **CRC_BG_3** groups.

Name	Pathway Name	Exact mass	P value	FC	logFC	Remarks
2-Methoxy-17beta-estradiol	Androgen and estrogen biosynthesis and metabolism	302.1883	6,59E-13	1,875365	-1,52725	negative value of logFC = lower metabolite content in CRC
24,25,26,27-tetranor-23-oxo-hydroxyvitamin D3	Vitamin D3 (cholecalciferol) metabolism	358.5143	1,91E-21	1,875365	-1,52725	negative value of logFC = lower metabolite content in CRC
4-Androsten-11beta-ol-3,17-dione	C21-steroid hormone biosynthesis and metabolism	302.1882	4,10E-02	2,064939	0,314907	
Acetyl-CoA	Aspartate and asparagine metabolism, Methionine and cysteine metabolism, Di-unsaturated fatty acid beta-oxidation, Leukotriene metabolism,	809.1258				too many metabolic pathways involved

	Fatty acid activation, Prostaglandin formation from arachidonate, Glycolysis and Gluconeogenesis, Pyruvate Metabolism, Squalene and cholesterol biosynthesis, Vitamin B1 (thiamin) metabolism, Histidine metabolism, Propanoate metabolism, Vitamin E metabolism, Glycine, serine, alanine and threonine metabolism, Tryptophan metabolism, Urea cycle/amino group metabolism, Valine, leucine and isoleucine degradation, Butanoate metabolism, Arginine and Proline Metabolism					
Androstenedione	C21-steroid hormone biosynthesis and metabolism	286.1933	4,10E-02	2,064939	0,314907	
cis,cis-myristo-5,8-dienoyl coenzyme A	Di-unsaturated fatty acid beta-oxidation		4,10E-03	39,0332	1,591434	
Dehydroepiandrosterone sulfate	C21-steroid hormone biosynthesis and metabolism	368.1657	4,10E-02	2,064939	0,314907	

Dehydroepiandrosterone sulfate	Androgen and estrogen biosynthesis and metabolism	368.1657	5,99E-05	1,895738	0,277778	
Elaidic acid	Fatty acid activation	282.2559	5,23E-06	3,75822		Metabolite absent in CRC_3BG
Glutathione disulfide	Aspartate and asparagine metabolism	612.152	3,68E-05	49,83188	1,697507	
Glutathione disulfide	Methionine and cysteine metabolism	612.152	3,68E-05	49,83188	1,697507	
Glutathione disulfide	Arachidonic acid metabolism	612.152	6,53E-07	2,76767		Metabolite absent in CRC_3BG
Glutathione disulfide	Linoleate metabolism	612.152	1,23E-02	1,851779	0,267589	
Glutathione disulfide	Ascorbate (Vitamin C) and Aldarate Metabolism	612.152	2,81E-03	0,029699	-1,52725	negative value of logFC = lower metabolite content in CRC
Oleic acid	De novo fatty acid biosynthesis	282.2559	6,59E-13	3,158659		Metabolite absent in CRC_3BG
Tamoxifen	Drug metabolism - cytochrome P450	371.2249	7,76E-03	1,636392	0,213887	
Uroporphyrin I	Porphyrin metabolism	830.2283	6,59E-13	3,158659		Metabolite absent in CRC_3BG
Uroporphyrin III	Porphyrin metabolism	830.2283	1,64E-14	3,080682		Metabolite absent in CRC_3BG
